# Reactive Oxygen Species Interact With NLRP3 Inflammasomes and Are Involved in the Inflammation of Sepsis: From Mechanism to Treatment of Progression

**DOI:** 10.3389/fphys.2020.571810

**Published:** 2020-11-25

**Authors:** Shuai Zhao, Fan Chen, Qiliang Yin, Dunwei Wang, Wei Han, Yuan Zhang

**Affiliations:** ^1^Department of Anesthesiology, First Hospital of Jilin University, Changchun, China; ^2^Department of Neurosurgery, University Medicine Greifswald, Greifswald, Germany; ^3^Department of Oncology, First Hospital of Jilin University, Changchun, China

**Keywords:** reactive oxygen species, inflammasome, sepsis, mitochondria, autophagy

## Abstract

Over the past 10 years, the crisis of sepsis has remained a great challenge. According to data from 2016, the sepsis-related mortality rate remains high. In addition, sepsis consumes extensive medical resources in intensive care units, and anti-inflammatory agents fail to improve sepsis-associated hyperinflammation and symptoms of immunosuppression. The specific immune mechanism of sepsis remains to be elucidated. Reactive oxygen species (ROS) are triggered by energy metabolism and respiratory dysfunction in sepsis, which not only cause oxidative damage to tissues and organelles, but also directly and indirectly promote NOD-, LRR-, and pyrin domain-containing protein 3 (NLRP3) inflammasome activation. NLRP3 inflammasomes enlarge the inflammatory response and trigger apoptosis of immune cells to exacerbate sepsis progression. Inhibiting the negative effects of ROS and NLRP3 inflammasomes therefore provides the possibility of reversing the excessive inflammation during sepsis. In this review, we describe the interaction of ROS and NLRP3 inflammasomes during sepsis, provide prevention strategies, and identify fields that need further study.

## Introduction

Sepsis is defined as a life-threatening organ dysfunction caused by infection (Singer et al., 2016). Based on worldwide data over the past decade, an estimated 31.5 million cases of sepsis and 19.4 million cases of severe sepsis require intensive treatment each year. The mortality rate is 17% in sepsis patients and 26% in severe sepsis patients (Fleischmann et al., 2016), rising to 80% in those patients who develop multiple organ failures (Marshall et al., 2005). When case mortality rates for sepsis in the hospital were used to estimate the global incidence, it was estimated that sepsis may contribute up to 5.3 million deaths per year (Fleischmann et al., 2016). It was the most expensive hospital treatment in the United States in 2014, with an average cost of more than $18,000 per stay (Chang D. W. et al., 2015). According to official statistics, sepsis accounts for more than $20 billion of hospital costs; this accounted for ∼5% of total hospital costs in the United States in 2011. However, with the extensive use of modern antibiotics in clinical practice, bacteriological theories cannot fully explain the pathogenesis of sepsis, because many sepsis patients die despite successfully eliminating the pathogen. Researchers have therefore proposed that it is the host, rather than bacteria, which exacerbates the inflammation of sepsis (Cerra, 1985).

Reactive oxygen species (ROS) are a group of molecules, which include peroxides, superoxide, hydroxyl radical, and singlet oxygen (Hayyan et al., 2016). ROS are produced in various biochemical reactions in cells and organelles, such as mitochondria, endoplasmic reticulum, and peroxisomes (Muller, 2000; Han et al., 2001). Physiological amounts of ROS are involved in maintaining homeostasis of the internal environment, including intracellular signaling, redox regulation (Ray et al., 2012), proliferation, host defenses, signal transduction, and mitochondrial biogenesis (Droge, 2002; Lee and Wei, 2005). In addition, ROS are also antimicrobial agents that can directly destroy microbial pathogens (Fang, 2011), while excess amounts can cause deleterious effects. During sepsis, excessive production of ROS causes significant cellular dysfunctions in organs, which contributes to the inability to treat sepsis, as well as multi-organ system failures (Fialkow et al., 2007; Hoetzenecker et al., 2011; Chen et al., 2017). In addition, ROS are known to promote apoptosis, mitochondrial oxidation, and alter cell signaling pathways.

The NOD-, LRR-, and pyrin domain-containing protein 3 (NLRP3) inflammasome is a multi-protein complex belonging to the inflammatory pathway of the innate immune system (De Nardo et al., 2014). It is composed of a NOD-like receptor (NLR), which initiates the signaling pathway, the adapter protein apoptosis-associated speck-like protein containing a caspase recruitment domain, and the executor protein, caspase-1 (De Nardo et al., 2014; He et al., 2016; Kelley et al., 2019). NLRP3 senses pathogen-associated molecular patterns (PAMPs) and damage-associated molecular patterns (DAMPs) to complete its priming and triggering (Kelley et al., 2019). The priming of NLRP3 inflammasome activation requires lipopolysaccharide/Toll-like receptor 4 (LPS/TLR4) signaling (Yang et al., 2019b). Then, the environmental stimuli mediate triggering, thereby promoting NLRP3 inflammasome assembly, and then caspase-1 processes the immature preforms of cytokines to mature cytokines (IL-1β and IL-18) (Bauernfeind et al., 2011; Franchi and Nunez, 2012). IL-1β and IL-18 induce a cascade of inflammatory responses during sepsis, leading to extensive inflammatory-associated damage and multi-organ dysfunctions (Sahoo et al., 2011). In addition, caspase-1-mediated pyroptosis of immune cells (Brodsky and Medzhitov, 2011; Miao et al., 2011) may lead to immune paralysis in the final stage of sepsis.

Reactive oxygen species interactions with NLRP3 inflammasomes participate in regulating the immune inflammation responses during sepsis. Studies have reported the mutually reinforcing effects of ROS and NLRP3 inflammasomes. (1) ROS generation partly depends on activation of NLRP3 inflammasomes (Petrilli et al., 2007; Dostert et al., 2008; Tschopp and Schroder, 2010). (2) ROS-mediated DAMPs are involved in activating NLRP3 inflammasomes to increase inflammation (Nakahira et al., 2011; Minutoli et al., 2016; Lee et al., 2017). 3) ROS-induced NLRP3 components may be important factors in triggering apoptosis of immune cells (Sarkar et al., 2006) during sepsis. However, other studies have suggested a different process involving upregulated autophagy activated by ROS, which may restrict NLRP3-mediated inflammation responses via eliminating the DAMPs/PAMPs. Considering the complicated interaction of ROS and NLRP3 inflammasomes, targeting the signal of ROS/NLRP3 inflammasomes may be a potential strategy for sepsis therapy. In this review, we mainly focused on the interaction of ROS and NLRP3 inflammasomes during sepsis, to describe the mechanism of inflammation as well as the progress in therapies.

## ROS Generation in Sepsis

### The Traditional Pathway of ROS Generation

Reactive oxygen species are a group of molecules that include oxygen radicals, as well as non-radicals (Russell and Cotter, 2015). The ROS produced by the respiratory chain located on the inner mitochondrial membrane during oxidative phosphorylation are the main sources of free radicals in most cell types (Turrens, 2003; Lee and Wei, 2005; Murphy, 2009; Li X. et al., 2017; Scialo et al., 2017; Reichart et al., 2019). Electrons leak from electron transport chains at the point of complex I and complex III, a process that results in the partial reduction of oxygen to form superoxide (Li X. et al., 2013; Kalogeris et al., 2014). Then, superoxide is rapidly converted to hydrogen peroxide via superoxide dismutase. Both superoxide and hydrogen peroxide are considered as ROS (Li X. et al., 2013). Furthermore, a previous study reported that calcium overload impaired mitochondrial function, leading to decreased ATP production and increased ROS generation in sepsis (Brookes et al., 2004; West et al., 2011; Feng et al., 2018). Ca^2+^ overload induced cytochrome c dislocation from the mitochondrial inner membrane, and increased ROS generation by blocking the respiratory chain at complex III (Grijalba et al., 1999; Ott et al., 2002). Based on these results, oxidative stress and intracellular calcium loading in mitochondria might explain the excessive ROS generation during sepsis. Most importantly, studies have reported an attenuation of endogenous antioxidants in sepsis patients (Donnino et al., 2011), so excessive ROS cannot be neutralized completely, which is also an important reason for its accumulation.

### ROS Generation Partly Depends on NLRP3 Inflammasomes

NLRP3 is a more general cellular stress sensor and its activation is accompanied by inflammasome activation dependent on ROS generation in spatial and temporal proximities (Petrilli et al., 2007; Zhou et al., 2010). LPS was reported to increase intracellular ROS levels by activating the TLR4 (West et al., 2011; Feng et al., 2018), a process known as priming the initiation phase of NLRP3 activation. The P2X7 receptor is a trimeric ATP-gated cation channel, with increased receptor membrane pore permeability leading to an efflux of K^+^ (Bartlett et al., 2014). It is well-known that ATP activates the NLRP3 inflammasomes via a feedback mechanism on the P2X7 channel and subsequently processes and releases IL-1β (Amores-Iniesta et al., 2017). Previous studies found that stimulation by ATP, asbestos, and silica promoted the generation of short-lived ROS (Petrilli et al., 2007; Dostert et al., 2008). ROS generation is frequently accompanied by K^+^ efflux (Kowaltowski et al., 2009). Low intracellular K^+^ concentration may therefore trigger ROS production in the process of NLRP3 inflammasomes activation, which suggests that ROS production may be dependent on the activation of the P2X7 receptor (Tschopp and Schroder, 2010). Future studies should focus on detailed studies involving the pathophysiological significance of ROS induced by the NLRP3-dependent pathway.

### ROS Promote NLRP3 Inflammasome Activation in Sepsis

Reactive oxygen species are potential signals for NLRP3 inflammasome activation. The exact mechanism of how ROS mediate NLRP3 inflammasome activation and assembly remains to be clarified. The potential mechanisms are summarized as follows.

Reactive oxygen species are sensed by a complex of thioredoxin and thioredoxin-interacting protein (TXNIP), and induce the dissociation of the complex. Under normal conditions, TXNIP is continuously connected to and retained in the reduced state by the ubiquitous TRX (Tschopp and Schroder, 2010). When the cellular ROS concentrations increase, the complex disengages and causes TXNIP to bind to the leucine-rich repeat of NLRP3, triggering activation of NLRP3 (Zhou and Chng, 2013).

Another potential mechanism involves mtDNA binding. Mitochondria are the sites of ROS production and the main targets of ROS attack. Excessive generation of mitochondrial ROS (mtROS) attacks DNA and generates oxidized bases and strand breaks (Maynard et al., 2009), which has a destructive effect on the electron transport chain (Cortes-Rojo and Rodriguez-Orozco, 2011; Galley, 2011; Guo et al., 2013). Finally, free mtDNAs are released into the cytoplasm to bind and activate NLRP3 (Nakahira et al., 2011; Lee et al., 2017). Microscopy has shown that mtDNA co-localizes with NLRP3, which results in their binding (Shimada et al., 2012). Normally, oxidation-induced DNA damage is corrected by base excision repair (Krokan and Bjoras, 2013). However, continuous oxidative stress causes a large amount of DNA damage, which may exceed the capacity of base excision repair, leading to accumulation of mtDNA mutations, which can constantly activate NLRP3 inflammasomes.

The third potential mechanism has some common steps with the second mechanism. The NLRP3 stimulators induce a special form of mitochondrial damage by increasing ROS production. Then, ROS converts mtDNA into an oxidative form to bind with NLRP3 (Shimada et al., 2012). This theory mainly emphasizes that NLRP3 stimulator-induced ROS generation may contribute to the oxidative form of mtDNA, which binds to NLRP3 for its activation.

Investigators have also suggested that mtDNA is insufficient to trigger NLRP3 signaling if the priming step is missing (Latz et al., 2013), which means that ROS-induced NLRP3 inflammasome activation also requires LPS/TLR4 participation as a priming signal.

IL-1β released by the mtROS/NLRP3 inflammasome pathway in platelets has been correlated with increased vascular permeability (Hottz et al., 2013), which may cause irreversible shock during sepsis. IL-1β-induced multiple organ damage in sepsis has also been reported (Kumar, 2018). In addition, ROS-induced mitochondrial destruction in sepsis will lead to decreased ATP production, resulting in dysfunction of immune cells and exacerbation of tissue hypoxia, all of which are reasons for immune disorder during sepsis.

### ROS-Induced Apoptosis Partially Depends on NLRP3 Inflammasomes

Excessive ROS trigger apoptosis, depending on extrinsic and intrinsic pathways (Gibson, 2010). In the extrinsic pathway, Fas ligand is involved in the production of ROS, which induces recruitment of the Fas-associated death domain, and subsequent induction of apoptosis (Gupta et al., 2012). The intrinsic pathway involves a caspase cascade, which is accompanied by oxidative stress, and activated by mitochondrial damage, resulting in release of damaged cytochrome c and DNA. This process is believed to promote proapoptotic protein expression and lead to apoptosis and death (Maiuri et al., 2007).

Apoptosis occurs during mitochondrial dysfunction. Highly expressed mtROS has been reported to promote apoptosis by opening mitochondrial permeability transition pores (mPTPs) during sepsis (Csordas et al., 1999; Durand et al., 2017). Cytochrome c and apoptosis-inducing factors released from mPTP contribute to ATP depletion and apoptosis (De Oliveira et al., 2006). In addition, NLRP3 inflammasome signaling has been proven to exacerbate cell apoptosis in sepsis-induced acute respiratory distress syndrome (Zhang et al., 2020). The mtDNA is released into the extracellular space, and oxidized mtDNA activates NLRP3 inflammasomes during apoptosis (Shimada et al., 2012). Caspase-1 triggers mitochondrial damage upon its activation by NLRP3 inflammasomes, which increases plasma membrane permeability (Yu et al., 2014), and makes the intima more permeable to small molecules, resulting in promotion of promote apoptosis.

Caspase-1 has been shown to promote splenic B cell apoptosis (Sarkar et al., 2006) and lymphocyte apoptosis during sepsis (Exline et al., 2014). High expression of caspase-1 was confirmed using immunohistochemistry in spleens from sepsis patients (Aziz et al., 2014). A large number of circulatory microvesicles (MVs) were identified in sepsis patients. When released by monocytes, MVs induced apoptosis in healthy donor lymphocytes, which greatly attenuated the apoptotic signals and strengthened protective immune responses against infection (Exline et al., 2014).

It has been shown that anti-apoptotic protein attenuates NLRP3-associated inflammation, which in turn reduces oxidized mtDNA to be released into the cytoplasm (Khare et al., 2012). Treatment with 8-hydroxy-guanosine decreases apoptosis-produced IL-1β triggered by NLRP3 inflammasomes. The mtROS may therefore oxidize guanosine residues, and cause release of a modified mtDNA to active NLRP3 inflammasomes (Khare et al., 2012). Although ROS and NLRP3/caspase-1-induced apoptosis in sepsis have been studied, future experiments are needed to show more evidence for their cooperation or interaction.

### ROS Activates Autophagy to Downregulate NLRP3 Inflammasomes

Although sufficient evidence has demonstrated negative effects of the ROS/NLRP3 inflammasome pathway during sepsis, some studies have also suggested an anti-inflammatory effect of ROS. Autophagy is thought to maintain cellular quality control to regulate innate immune responses, and is therefore adaptive under physiological conditions or mild stress. Autophagy provides a degree of control to promote survival and maintain normal functioning of organs. It is speculated that autophagy is adaptive in the early stages of sepsis. However, when sepsis progresses to a more severe condition, the autophagy response is incapable of completing its function, which leads to unadaptive results (Sun et al., 2019).

Reactive oxygen species have many times been reported as early inducers of autophagy during low nutrient stress (Filomeni et al., 2010). AMP-activated protein kinase (AMPK) contributes to autophagosome maturation (Jang et al., 2018). ROS can regulate autophagy through AMPK and nutrient deprivation, and induction of AMP contributes to autophagy induction (Inoki et al., 2003; Gwinn et al., 2008). Nutritional disorder accompanied by excessive ROS generation may therefore be a trigger for increasing the activity of autophagy during sepsis. ROS can also regulate autophagy by increasing the activity of transcription factors such as NF-κB, to induce autophagy gene expression (Djavaheri-Mergny et al., 2006; Chen et al., 2008). Furthermore, ROS destroy mitochondrial integrity during sepsis, and damage of the mitochondrial membrane is also one of the signals for the initiation of autophagy (Lyamzaev et al., 2018).

Macrophages show more dysfunctional mitochondria and mtDNA accumulation when under-stimulated by LPS and ATP after depletion of autophagic proteins (Nakahira et al., 2011). Over-accumulation of circulated mtDNA has a high correlation with increased mortality in the intensive care unit. The mtDNA serves as a viable plasma biomarker for critically ill patients (Nakahira et al., 2013; Johansson et al., 2018), and activates NLRP3 to trigger cytokine release and exacerbate the inflammatory response of sepsis. Through the selective elimination of mtDNA and mtROS, autophagy has been seen as a key regulator in maintaining the stability of mitochondria (Nakahira et al., 2011), which helps to control NLRP3 inflammasome-associated hyper-inflammatory responses (Kim et al., 2016b). In addition, cytosolic mtDNA fragments also stimulate inflammation signals like TLR9 and cyclic GMP–AMP synthase, to aggravate the inflammatory response of sepsis (Schafer et al., 2016; West and Shadel, 2017; Hu et al., 2019), which emphasizes the significant role of autophagy in clearing DNA during the development of sepsis.

**FIGURE 1:**
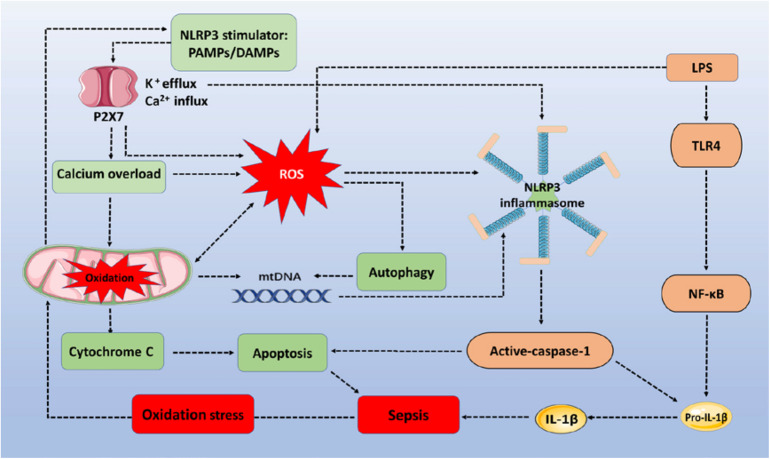
The ROS/NLRP3 signaling pathway participates in sepsis-related immune responses. The sepsis associated oxidative stress induces ROS generation via oxidation of mitochondria. PAMPs/DAMPs trigger P2X7 activation and promote Ca^2+^ influx and K^+^ efflux. Increased Ca^2+^ influx (calcium overload) and ROS destroy the stability of mitochondria, triggering mtDNA and cytochrome C accumulation within the cytoplasm, to assemble NLRP3 inflammasomes and induce apoptosis. LPS activates TLR4/NF-κB signaling to express pro-IL-1β and pro-IL-18. Activated caspase-1 then cleaves pro-IL-lβ and pro-IL-18 into their mature forms to increase the inflammatory response. In addition, NLRP3 inflammasomes depend on caspase-1 to mediate immune cell apoptosis. During the whole process, autophagy is activated by ROS to clear mtDNA, and to partially reduce NLRP3 inflammasome activation. ROS, reactive oxygen species; NLRP3, NOD-, LRR-, and pyrin domain-containing protein 3; TLR4, toll-like receptor 4; LPS, lipopolysaccharides; PAMPs/DAMPs, pathogen-associated molecular patterns and damage-associated molecular patterns; mtDNA, mitochondrial DNA.

However, ROS upregulation of autophagy to inhibit inflammatory responses has limitations. Upregulated autophagy may be insufficient to counteract the NLRP3 inflammasome-induced negative effects of inflammation increases during sepsis. First, autophagy decreases the activation of nucleic acid recognition receptors, and clearance of damaged mitochondria may only occur in the early stage of sepsis, before autophagic proteins are consumed. For example, a recent study showed that the severity of sepsis determines the activity of autophagy. When given a low dose of LPS, to create a model of mild sepsis, autophagy activity was increased proportionately to the degree of damage. However, in the case of severe sepsis, autophagy was not proportional to the degree of damage (Sun et al., 2018). This indicates that the increased autophagy demand in severe sepsis cannot meet the requirements of maintaining homeostasis of the internal environment. In addition, upregulated autophagy mainly reduces the inflammatory responses from nucleic acid recognition receptors, but fails to effectively eliminate inflammation from other signal pathways. It is generally accepted that inflammatory signal receptors sense a variety of stimulators for their activation during sepsis. However, reducing autophagy protein consumption or improving autophagy protein activity should provide new ideas and research directions for sepsis therapy. Figure 1 describes how the ROS/NLRP3 signaling pathway regulates cell apoptosis, inflammatory responses, autophagy, and mitochondrial injury in sepsis progression.

## Treatment Strategies

**FIGURE 2:**
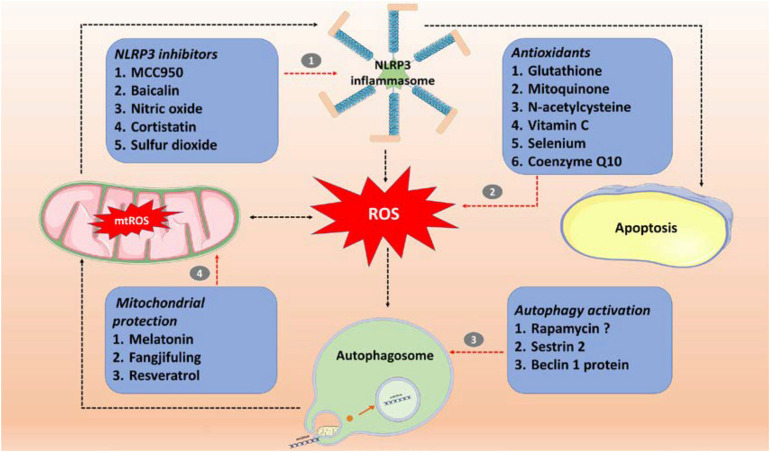
Sepsis treatment involves targeting the ROS/NLRP3-centered inflammatory pathway. (1) Specific inhibitor (MCC950) and non-specific inhibitors (baicalin, nitric oxide, cortistatin, and sulfur dioxide) of NLRP3 inflammasomes; (2) antioxidants (glutathione, mitoquinone, *N*-acetylcysteine, vitamin C, selenium and coenzyme Q10); (3) autophagy activators (rapamycin, SESN2, and beclin 1 protein); (4) mitochondrial protection (Fangjifuling and resveratrol). ROS, reactive oxygen species; mtROS, mitochondrial reactive oxygen species; NLRP3, NOD-, LRR-, and pyrin domain-containing protein 3.

The germ theory and antibiotic therapy have limitations. Prevention of sepsis by switching from hyper-immunity to immunosuppression is the key to sepsis treatment, because the latter stage has a high rate of mortality. Based on the known immune injury mechanisms, investigators have used a series of sepsis treatment options, which have been partially successful in both animal experiments and clinical trials. Figure 2 and Table 1 summarize treatments that involve inhibiting ROS and NLRP3 inflammasome production, increasing the activity of autophagy, and maintaining the stability of mitochondria.

**TABLE 1:** Evidence for sepsis treatment strategies by targeting NLRP3, ROS, autophagy, and mitochondria.

Target point	Compound	Function or mechanism	References
NLRP3 inhibitors	MCC950	MCC950 inhibits NLRP3 inflammasomes to reduce septic death in mice.	Kang et al., 2016; Lin et al., 2019
	Baicalin	Baicalin decreases NLRP3 inflammasome activation to improve survival in sepsis mice by augmenting protein kinase A signaling.	Li C. G. et al., 2017
	Cortistatin	Cortistatin deactivates NLRP3 inflammasome activity to reduce myocardial injury induced by sepsis.	Zhang B. et al., 2015
	Sulfur dioxide	Sulfur dioxide attenuates sepsis-induced cardiac dysfunction by inhibiting inflammation via the TLR4/NLRP3 signaling pathway.	Yang et al., 2018
ROS scavengers	Glutathione	Glutathione exhibits a protective effect by removing excess reactive oxygen species (ROS) in sepsis mice.	Pompella et al., 2003; Zhou et al., 2016; Yang et al., 2019a
	Mitoquinone	Mitoquinone reduces ROS formation to increase the survival rate of mice challenged by LPS.	Lowes et al., 2008; Ramsey and Wu, 2014
Antioxidants (clinical trial)	*N*-acetylcysteine	*N*-acetylcysteine improves liver function, respiratory function, and reduces ICU therapy stays for sepsis patients.	Spapen et al., 1998; Rank et al., 2000; Paterson et al., 2003
	Vitamin C	Vitamin C reduces sequential organ failure assessment score, C-reactive protein and procalcitonin, and prevents colistin associated acute kidney injury.	FowlerIII, Syed et al., 2014; Dalfino et al., 2015;
	Selenium	Selenium reduces inflammation biomarkers, and predicts and decreases infection-related hospital mortality.	Forceville, 2007; Valenta et al., 2011; Costa et al., 2014
	Coenzyme Q10	Coenzyme Q10 reduces inflammatory biomarkers and improves survival of sepsis patients.	Soltani et al., 2020
Autophagy activators	Sestrin 2	Sestrin 2 induces mitophagy to clean damaged mitochondria and inhibit NLRP3 activation in sepsis.	Kim et al., 2016a
	Beclin 1 peptide	Beclin-1 protects mitochondria to reduce the release of mitochondrial danger-associated molecular patterns, and promotes mitophagy to decrease sepsis.	Sun et al., 2018
Mitochondrial protectants	Fangjifuling	Fangjifuling suppresses mitochondrial-mediated apoptosis by regulating the Bax/Bcl-2 ratio in LPS-induced inflammatory.	Su et al., 2018
	Resveratrol	Resveratrol attenuates widespread swollen mitochondria with ruptured outer membranes to reverse sepsis-induced myocardial remodeling.	Smeding et al., 2012

*NLRP3, NOD-, LRR-, and pyrin domain-containing protein 3; TLR4, Toll-like Receptor 4; Bax/Bcl-2, Bax (apoptosis inducer), Bcl-2 (apoptosis suppressor); LPS, lipopolysaccharides.*

## Antioxidant Usage

Reactive oxygen species destroy mitochondrial integrity, promote cell apoptosis, and aggravate the inflammatory responses by promoting NLRP3 inflammasome activation in sepsis (Brealey et al., 2002; Nakahira et al., 2011; Lee et al., 2017; Oliveira et al., 2017; Li et al., 2018). Strategies have focused on eliminating ROS to reduce mitochondrial damage in sepsis. ROS scavengers and antioxidants have already been shown to decrease NLRP3 activation (Petrilli et al., 2007; Dostert et al., 2008).

It has been reported that ROS derived from nicotinamide adenine dinucleotide phosphate oxidase inhibits LPS-induced acute inflammatory responses, suggesting that this enzyme induces ROS, which may limit the cytotoxicity and alterations of reduction reactions such as changes in reduced expression of glutathione (GSH) levels during sepsis (Zhang et al., 2009; de Jong et al., 2010; Huet et al., 2011). Glutathione is an antioxidant, which prevents oxidative damage by ROS (Pompella et al., 2003). GSH scavenges ROS and converts harmful hydrogen peroxide to water, which reduces mitochondrial damage (Zhou et al., 2016; Yang et al., 2019a). Selenium is an essential trace metal, which participates in the process of antioxidant as a result of its incorporation into glutathione peroxidases (Holben and Smith, 1999). Adequate supplementation of trace elements in sepsis may therefore contribute to recovery. In addition, interference with GSH synthesis exacerbates sepsis-associated acute lung injury (Qian et al., 2018). Enhancing glutathione endogenous synthesis and exogenous administration will therefore facilitate increased resistance against the excessive accumulation of free radicals in sepsis.

Mitoquinone (MitoQ) is one of the most studied mitochondrial-targeted antioxidants, which has been reported to preserve mitochondrial dysfunction during heart failure (Ribeiro Junior et al., 2018), and significantly increase the survival rate of sepsis mice (Lowes et al., 2008; Ramsey and Wu, 2014). MitoQ has also been reported to suppress NLRP3 inflammasome-mediated inflammatory cytokines (Dashdorj et al., 2013), which may be related to its mitochondrial protection effect. Coenzyme Q is a unique lipid-soluble antioxidant and a mobile component of the mitochondrial electron transport chain (Laredj et al., 2014; Wang and Hekimi, 2016). MitoQ is a patented and scientifically validated “super” version of coenzyme Q, which means that it has similar anti-inflammatory effects.

NLRP3 activators and the P2X7 receptor are involved in NLRP3 activation and short-lived ROS production (Petrilli et al., 2007; Dostert et al., 2008; Tschopp and Schroder, 2010). Clearance of NLRP3 activators or inhibiting the P2X7 receptor could be a strategy to reduce ROS production and further inhibit NLRP3 inflammasomes activation. In addition, extensive ROS and damaged mitochondrial may also induce apoptosis (Morgan et al., 2007), suggesting that scavenging of ROS and mitochondrial protection using antioxidants may be an effective treatments for sepsis.

## Activation of the Autophagy System

Improved autophagic activity is beneficial in controlling inflammation responses by maintaining the stability of mitochondrial quality control (Nakahira et al., 2011). For these reasons, mitophagy activation may be a possible therapeutic adjunct to reverse sepsis (Chang A. L. et al., 2015; Jin et al., 2017). However, autophagy-based therapies are currently not available for clinical use.

Rapamycin is an inhibitor of the mammalian target of rapamycin (mTOR). A recent study showed its ability to induce autophagy (Laplante and Sabatini, 2012). It played an active role in improving the symptoms of senility diseases such as Alzheimer’s disease (Harrison et al., 2009) and sepsis-induced cognitive impairment (Liu et al., 2017). However, mTOR also plays an important role in maintaining internal environmental homeostasis (Laplante and Sabatini, 2012). Therefore, the therapeutic efficiency of inhibiting mTOR by rapamycin in sepsis mice is highly uncertain.

Various proteins have been shown to maintain the stability of autophagy (Wei et al., 2008; Zhang Q. et al., 2015; Kim et al., 2016a; Sun et al., 2018). Sestrin 2, known as stress-inducible protein, suppresses NLRP3 inflammasome activation by inducing the activity of mitophagy, to eliminate damaged mitochondria in macrophages (Kim et al., 2016a). The Beclin 1 protein, a central regulator of autophagy in mammalian cells, has shown therapeutic effects for sepsis. Injection of the cell permeable Tat-Beclin-1 peptide has been shown to improve the cardiac function of sepsis mice (Sun et al., 2018, 2019). Although increased activation of autophagy contributes to decreased inflammation responses in sepsis, we cannot exclude whether it also increases the risk of clearing normal mitochondria; this negative effect should also be considered.

## NLRP3 Inflammasome Pathway Inhibitors

Inhibition of NLRP3 inflammasomes has shown great benefit for inflammation control and life extension in sepsis mice (Lee et al., 2017; Song et al., 2020). The entire process of NLRP3 inflammasome activation requires participation of both priming and triggering signals, so their inhibitions may be beneficial for controlling the inflammation response in sepsis. The TLR4 inhibitors, eritoran, polyamidoamine, and tak-242, have been shown to decrease the inflammatory response in sepsis animals (Fenhammar et al., 2011; Kalil et al., 2011; Teo et al., 2012), and the NLRP3 inhibitor, MCC950, has been shown to have a positive effect in sepsis mice (Kang et al., 2016; Lin et al., 2019). In addition, non-specific inhibitors including baicalin, cortistatin, and sulfur dioxide have been shown to alleviate the inflammatory response by inhibiting NLPR3 inflammasomes in sepsis mice (Zhang B. et al., 2015; Li C. G. et al., 2017; Yang et al., 2018). NLRP3 inhibition cannot only reduce the levels of inflammatory factors such as IL-1β and IL-18, but it can also reduce oxidative stress and cell apoptosis. NLRP3 inhibitors therefore have advantages in the possible treatment of sepsis.

## Mitochondrial Protection

The mitochondrial membrane is a structure, which maintains mitochondrial stability. Swollen mitochondria with ruptured outer membranes are characteristic of sepsis (Zhang et al., 2018). Triggered by apoptosis, BAX and BAK form stomata on the mitochondrial membrane, which enables mtDNA release to further enlarge inflammatory responses (McArthur et al., 2018; Riley et al., 2018; Vringer and Tait, 2019).

Melatonin administration decreases the septic response, reduces inflammation and oxidative stress, and mitigates mitochondrial malfunction (Leon et al., 2005; Zhang et al., 2013; Volt et al., 2016). Melatonin scavenges oxygen and nitrogen reactants in mitochondria, which reduces the damage of mtDNA and mitochondrial protein. Melatonin also increases the activity of the electron transport chain at the point of complexes I and IV, thereby improving the function of the mitochondrial respiratory chain and increasing the efficiency of ATP synthesis (Leon et al., 2005).

MitoQ and coenzyme Q are orally active antioxidants that could target mitochondrial dysfunction to balance the oxidative damage and protect the mitochondria. We have reviewed their function and mechanism in the antioxidants section of this review.

Traditional Chinese medicines like Fangjifuling and resveratrol are believed to maintain the stability of mitochondrial membranes in a non-specific manner. Fangjifuling has been confirmed to ameliorate LPS-induced renal injury via inhibition of inflammatory and apoptotic responses in mice, and to suppress mitochondrial-mediated apoptosis by regulating the levels of anti-apoptotic proteins (Su et al., 2018). Resveratrol has been reported to significantly attenuate swollen mitochondria with ruptured outer membranes during sepsis (Smeding et al., 2012). In conclusion, maintaining mitochondrial membrane stability is beneficial for sepsis treatment by decreasing apoptosis and inflammatory signal activation. However, more drugs targeting mitochondrial membranes need to be identified.

## Progress in Clinical Treatment

Although animal experiments have been reported, numerous indirect schemes have been developed to reduce oxidative-associated damage, such as mitochondrial protection, activation of the autophagy system to eliminate ROS, and inhibition of NLRP3 inflammasomes. However, considering the potential negative effects, only some substances with antioxidant properties have been used in clinical trials. Clinical data showed decreased expression of antioxidants in patients with sepsis, which may be related to the inflammatory cascade of septic shock (Donnino et al., 2011). Excess oxidative stress consumes a large amount of endogenous antioxidants in septic patients, so appropriate exogenous administration of antioxidants may help control oxidation-associated inflammatory responses. Antioxidants are widely used in clinical practice to improve the outcomes of sepsis or to assist in the treatment of sepsis. However, some studies have suggested a lack of efficacy and even negative effects, which have hindered the approval of future clinical applications.

## *N*-Acetylcysteine

*N*-acetylcysteine (NAC), an antioxidant sulfhydryl molecule that has shown efficacy in sepsis therapy, is a precursor in the formation of GSH in the body. NAC increased liver blood flow and improved liver function in septic shock patients (Rank et al., 2000). Short-term NAC infusion was well-tolerated, and in patients with early septic shock, it not only improved respiratory function, but also shortened stays in the ICU (Spapen et al., 1998). The protective effects of NAC in sepsis results from its inhibition of NF-κB activation (Paterson et al., 2003), which is an essential priming step for NLRP3 inflammasome activation.

However, there are some negative reports involving its clinical use. For example, it was reported that high doses of NAC not only failed to improve patient outcomes, but also increased the risk of inflammation, and was associated with increased serum creatinine (Najafi et al., 2014). Early NAC administration failed to influence the microalbuminuria/creatinine ratio in severe clinical sepsis, suggesting that NAC might be ineffective in alleviating endothelial injury. NAC treatment even aggravated sepsis-induced cardiovascular failure (Spapen et al., 2005). The cardiovascular sequential organ failure assessment score progressively increased during NAC treatment, when compare with the control group (Spapen et al., 2005). In addition, NAC augmented neutrophil phagocytosis, which may also be detrimental to septic patients (Heller et al., 2001).

## Vitamin C (Sodium Ascorbate)

Vitamin C is a powerful antioxidant with the ability to donate a hydrogen atom and form a relatively stable ascorbic-free radical, which provides protection against oxidative stress-induced cellular damage by scavenging ROS (Traber and Stevens, 2011). Vitamin C administration also protects against oxidative damage, and shows an auxiliary effect on sepsis progression; patients receiving ascorbic acid exhibited prompt reductions in sequential organ failure assessment score. Ascorbic acid significantly reduces the proinflammatory biomarkers, C-reactive protein and procalcitonin (FowlerIII, Syed et al., 2014). Patients with severe sepsis may also need high doses of colistin to protect against drug-resistant Gram-negative bacteria. The most notable adverse effect of colistin is renal function impairment, especially with a significant increase of acute kidney injury in older patients. Administration of ascorbic acid significantly reduced the incidence of acute kidney injury, and facilitated the safer use of colistin (Dalfino et al., 2015).

Some studies have reported the ineffectiveness of vitamin C in the treatment of infectious diseases. For example, a clinical trial showed that a 96 h infusion of vitamin C did not result in an improvement of the sequential organ failure assessment score, or alter biomarkers of inflammation and vascular injury in sepsis patients (FowlerIII, Truwit et al., 2019). Similar studies also showed that ascorbic acid did not result in a statistically significant reduction in sequential organ failure assessment score during the first 72 h in patients with septic shock (Moskowitz et al., 2020). At least the previous clinical data did not reveal a negative or harmful effect of vitamin C, which indicates the safety of its use. Moreover, it is controversial whether to evaluate the therapeutic effect of vitamin C using a simple sequential organ failure assessment score or proinflammatory biomarkers. Further research is expected to evaluate the potential role of vitamin C in sepsis from the perspective of its antioxidant effects, mitochondrial protection, and alterations of inflammasomes.

## Selenium

Selenium is an essential anti-oxidant required for anti-oxidant seleno-enzymes. The erythrocyte selenium concentration was lower in patients who died in the ICU, which may help to predict infection-related hospital mortality (Costa et al., 2014). A bolus administration of high dose selenium has been associated with a tendency to decrease mortality in septic shock patients (Forceville, 2007). However, the study stated that high dose selenium substitution in patients with sepsis failed to reduce mortality, but it did reduce levels of inflammatory biomarkers (Valenta et al., 2011).

## Coenzyme Q10

Animal experiments have widely reported the antioxidant effect of Coenzyme Q10 in septic mice. Coenzyme Q10 levels were expressed at low levels, and were associated with the inflammatory cascade in septic shock, which indicated that it was negatively associated with vascular endothelial markers and inflammatory molecules (Donnino et al., 2011). Soltani et al. (2020) reported beneficial effects of Coenzyme Q10 on survival and inflammatory marker reduction in septic patients. Another clinical study reported that oral Coenzyme Q10 increased the plasma concentration of Coenzyme Q10, but had no effect on clinical outcomes (Donnino et al., 2015).

## Melatonin

Serum melatonin level was positively associated with serum levels of biomarkers of oxidative stress and sequential organ failure assessment score (Lorente et al., 2015), and was therefore associated with sepsis severity and mortality (Lorente et al., 2018). A clinical trial involving 20 healthy participants showed the safety of oral melatonin in doses of 20–100 mg, with no adverse effects other than mild transient drowsiness with no effects on sleeping patterns (Galley et al., 2014). Melatonin administration before endotoxemia resulted in reduction of inflammation and oxidative stress (Alamili et al., 2014b). In addition, melatonin also improved the clinical outcomes of neonatal sepsis, based on the observations of sepsis-related serum parameters(Gitto et al., 2001). Moreover, there was significantly decreased mortality in the melatonin therapy group in children, when compared to the control group. However, a limitation of these trials was the lack of sufficient numbers of patients. However, physiological secretion of melatonin occurred primarily at night, so its therapeutic effects may have been time-dependent. Melatonin had no beneficial effect on inflammation and oxidative damage caused by nocturnal endotoxemia, when compared with daytime endotoxemia (Alamili et al., 2014a), so the appropriate administration time may be important in melatonin therapy.

Although there is some controversy about the efficacy of antioxidants in the treatment of sepsis, some combination therapies have been reported to have clinical benefits. For example, combined infusion of vitamin C and NAC improved patient clinical scores in the ICU at day 5, and ultimately decreased patient mortality (Sandesc et al., 2018). In another study, administration of NAC plus GSH significantly decreased the oxidative stress of patients with septic shock (Ortolani et al., 2000). Based on these results, we hope that antioxidants will be successful in clinical trials as an adjunct to sepsis treatment.

## Discussion

Sepsis remains a great challenge for public health. In sepsis, oxidative stress responses are increased, followed by ROS over-production, which destroys the integrity of mitochondria and leads to the disorders of energy metabolism. NLRP3 then sense the mtDNA to finish the activation process. Inflammasomes and mitochondria dysfunction are involved in the induction of cell apoptosis. Finally, ROS, NLRP3 inflammasomes, mitochondrial damage, and cell apoptosis form a vicious cycle, leading a second strike on homeostasis, which is independent of bacteria. This second strike by immune inflammation may be the reason for irreversible immunosuppression in the late stages of sepsis, which cause high mortality even under adequate life support in the intensive care unit.

Reactive oxygen species act as bactericides, to protect the body from invasion by external microorganisms (Buchon et al., 2013). In addition, as a secondary signal, ROS is also involved in multifunctional protein expression and intracellular signal transduction (Ghosh and Mitchell, 1999). While excessive ROS are involved in disrupting the physiological balance. ROS and mtDNA serves as danger signal of sepsis progression in the ICU, and their dynamic monitoring is helpful in assessing the immune function and mortality of sepsis.

It is well-known that sepsis is characterized by hyperactive inflammation in the early stages and irreversible immunosuppression in the late stages. Future studies should therefore address the roles of ROS and NLRP3 inflammasomes in various pathophysiological and immunology stages in sepsis, to identify ways to manipulate mitochondrial damage and/or mitophagy as a means of modulating apoptosis and inflammatory responses.

BRCA1/BRCA2-containing complex 3 is a lysine 63-specific deubiquitinating enzyme involved in inflammasome activity, interferon signaling, and DNA damage repair (Meyer et al., 2020). A previous study showed that de-ubiquitination of NLRP3 by BRCA1/BRCA2-containing complex 3 critically regulated inflammasome activity (Py et al., 2013), with oxidative deactivation of specific enzymes by oxidation of co-factors. We are therefore investigating whether ROS influences the activity of NLRP3 through the oxidation of ubiquitination enzymes.

It is important to note that mtDNA cannot only be sensed by NLRP3, but also by multi-signaling pathways, such as TLR9 and cyclic GMP-AMP synthase, to induce the progression of sepsis (Schafer et al., 2016; West and Shadel, 2017; Hu et al., 2019). In addition, absent in melanoma 2, the NLR family CARD domain containing 4 and NLRP1 inflammasomes were able to recognize nucleic acids and activate their downstream products, caspase-1 and IL-1β (Sorbara and Girardin, 2011; Bae et al., 2019). The active expression of numerous nucleic acid recognition receptors in sepsis highlights the potential therapeutic significance of mitochondrial protection. Because this review mainly introduced the potential association of ROS-NLRP3 during sepsis, we have reduced the length of the chapter to introduce information regarding other inflammasomes.

Theoretically, ROS associated immuno-inflammatory responses can be treated by antioxidants. But recent studies show limited results in both animal experiments and clinical trials. Antioxidants have been used as adjuvant drugs in clinical studies. However, their therapeutic effects failed to receive extensive recognition by clinical doctors and their therapeutic uses are still being debated. Therefore, we may not expect to completely reverse the immune damage of sepsis only by antagonism of ROS, but should pay more attention to a comprehensive therapy management.

In summary, sepsis is a disorder with complex immune inflammatory responses involving ROS as the key trigger in the activation of NLPR3 inflammasomes in sepsis. Blocking ROS/NLPR3 inflammasome signals may provide the possibility to reverse sepsis.

## Author Contributions

SZ, YZ, and WH: conceptualization. DW and WH: supervision. SZ and FC: writing – original draft. YZ, WH, and QY: modification.

## Conflict of Interest

The authors declare that the research was conducted in the absence of any commercial or financial relationships that could be construed as a potential conflict of interest.
